# Treatment of thin endometrium with autologous platelet-rich plasma: a pilot study

**DOI:** 10.5935/1518-0557.20170013

**Published:** 2017

**Authors:** Shahrzad Zadehmodarres, Saghar Salehpour, Nasrin Saharkhiz, Leila Nazari

**Affiliations:** 1 Department of Obstetrics and Gynecology, Preventive Gynecology Research Center, Shahid Beheshti University of Medical Sciences, Tehran, Iran

**Keywords:** platelet-rich plasma, thin endometrium, frozen-thawed, embryo transfer

## Abstract

Endometrium is one of the main factors in pregnancy. During assisted reproductive technology (ART) treatments, some cycles are cancelled due to inadequate endometrial growth. This study was conducted to evaluate the effectiveness of platelet-rich plasma (PRP) in the treatment of thin endometrium. Ten patients with history of inadequate endometrial growth in frozen-thawed embryo transfer (FET) cycles were recruited into the study. Intrauterine infusion of PRP was performed. Endometrial thickness was assessed. Chemical and clinical pregnancies were reported. In all patients, endometrial thickness increased after PRP and embryo transfer was done in all of them. Five patients were pregnant. According to this study, it seems that PRP was effective for endometrial growth in patient with thin endometrium.

## INTRODUCTION

Endometrium is one of the main factors in implantation and pregnancy. Pregnancy rate is increased with growing endometrial thickness. In several studies, the minimum endometrium thickness for embryo transfer was reported to be 7 mm (El-Toukhy *et al.*, 2008; Richter *et al.*, 2007). Several methods are performed for endometrial preparation in frozen-thawed embryo transfer (FET) cycles, and there is little consensus on the most effective route. Some FET cycles are cancelled due to thin endometrium despite routine treatment, and there is no established protocol for this condition. Extended estrogen treatment and adjuvant therapy, such as low dose Aspirin, vaginal Sildenafil, Pentoxifylline and intrauterine perfusion with granulocyte-colony stimulating factor (G-CSF) have been used for thin endometrium, but there isn't any proved evidence in this treatment (Barad *et al.*, 2014; Chang *et al.*, 2015; Eftekhar *et al.*, 2014; Gleicher *et al.*, 2013; Groenewoud *et al.*, 2013; Lebovitz & Orvieto, 2014; Xu *et al.*, 2015).

Intrauterine infusion of platelet-rich plasma (PRP) is a new approach that has been suggested for the treatment of thin endometrium (Chang *et al.*, 2015). PRP is blood plasma prepared from fresh whole blood that has been enriched with platelets. It is collected from peripheral veins and contains several growth factors such as vascular endothelial growth factor (VEGF), epidermal growth factor (EGF), platelet derived growth factor (PDGF), transforming growth factor (TGF) and other cytokines that stimulate proliferation and growth. Recently, PRP has been used in several medical conditions in ophthalmology, orthopedics, surgery and wound healing but it's efficacy in endometrial growth has not been fully elucidated. The aim of this study was to evaluate the effectiveness of intrauterine infusion of PRP in the treatment of thin endometrium in FET cycles (El-Anwar *et al.*, 2016; Lee *et al.*, 2016; Maria-Angeliki *et al.*, 2015; Picard *et al.*, 2015; Ronci *et al.*, 2015; Rossi *et al.*, 2016; Sadabad *et al.*, 2016).

## CASES DESCRIPTION

Ten patients who had a history of cancelled cycles due to inadequate endometrial growth (less than 7 mm) in the past FET cycles despite standard treatments, were recruited into the study performed in the IVF center, Taleghani Hospital, Tehran, Iran from September 2015 to May 2016. All patients signed an informed written consent. The study was approved by the ethical committee of the Shahid Beheshti University of Medical Sciences (SBMU) (IR.SBMU.SM.REC.1394.92). Patients' characteristics and FET outcome data are summarized on Table 1.

**Table 1:** Patients' characteristics and FET outcomes

Patient	Age	Diagnosis	Endometrial* thickness (mm)	Embryos transferred (no.)	Chemical pregnancy	Clinical pregnancy
1	30	Tubal factor	4/6.1/7.2	2	No	No
2	34	Male Factor	5/6.8/7.5	3	Yes	Yes
3	33	Anovulation + Male factor	5/6/7.1	2	No	No
4	30	Endometriosis + Male factor	4.5/5.8/7.5	2		
5	39	DOR**	4.6/5.2/7.2	2	Yes	No***
6	35	DOR**	4.5/5.5/7	3	No	No
7	31	Male factor	6/6.3/7.4	2	Yes	Yes
8	39	DOR** + Male factor	4.3/5.2/7.1	2	Yes	Yes
9	35	Anovulation + Male factor	4.8/5.2/7.2	2	No	No
10	37	DOR	5.3/6.1/7.3	2	Yes	Yes

* Endometrial thickness (mm): Before PRP/48 h after first PRP/48h after second PRP

** DOR: Diminished ovarian reserve

*** Miscarriage

Hysteroscopic examination was performed before the cycle, if it had not been done previously. Hormone replacement therapy (HRT) was performed for endometrial preparation in all participant: estradiol valerate (Progynova; Bayer Schering Pharma, France) 6 mg/d was started on the 2^nd^ or 3^rd^ day of the mensural cycle and it was increased to 8 mg/d on day 9-10 because of inadequate endometrial growth (< 7 mm). PRP was performed on day 11-12 in all the patients due to thin endometrium and it was repeated on day 13-14. During the cycle, whenever the endometrial thickness was more than 7 mm, suppository progesterone (Cyclogest; Actavis, UK limited, England) 400 mg twice-a-day was started and embryo transfer (ET) was carried out per embryonic stage. Estradiol valerate and progesterone supplementation were continued for 2 weeks after ET and if the serum βHCG was positive, hormone supplementations were continued until 12 weeks of gestation.

Transvaginal ultrasound was performed by an expert gynecologist with a fellowship in infertility by one machine. Endometrial thickness was measured at the thickest part in the longitudinal axis of the uterus.

PRP was prepared from autologous blood using a two-step centrifuge process. On the 9^th^ or 10^th^ day of the mensural cycle, 17.5 ml of peripheral venous blood was drawn in the syringe that contained 2.5 ml of Acid Citrate A Anticoagulant solution (ACD-A) (Arya Mabna Tashkhis, Iran) and centrifuged immediately at 1200 rpm for 12 min to separate the red blood cells. The plasma was centrifuged again at 3300 rpm for 7 min to obtain the PRP. Then, 0.5 ml of PRP was infused into the uterine cavity with the IUI catheter (Takwin, Iran).

The primary outcome was endometrial expansion and the secondary outcomes were chemical and clinical pregnancies, determined by positive serum βHCG, 2 weeks after ET and the presence of fetal hear beat in the transvaginal ultrasound 5 weeks after ET.

## RESULTS

A total of 10 patients with a history of FET cancellation due to thin endometrium were recruited into the study. Uterine cavity abnormalities were not detected before starting the cycle. Four participants had a past of therapeutic resectoscopic hysteroscopy due to Asherman's syndrome and myoma. All the participants needed PRP in the treatment cycles due to inadequate endometrium growth. Endometrial thickness increased at 48 h after the first PRP and reached more than 7 mm after the second PRP in all patients. Embryo transfer was then carried out for all of them. Five patients were pregnant and in four of them the pregnancy progressed normally.

## DISCUSSION

PRP is autologous blood plasma that has been enriched with platelets at about 4-5 times more than the circulating blood. PRP can stimulate proliferation and regeneration with a large amount of growth factors and cytokines, including PDGF, TGF, VGEF, EGF, fibroblast growth factor (FGF), insulin-like growth factor I, II (IGF I, II), interleukin 8 (IL-8) and connective tissue growth factor (CTGF). Currently, PRP infusion is being increasingly used in several fields in medicine such as nerve injury, osteoarthritis, chronic tendinitis, bone repair and regeneration, cardiac muscles, alopecia, plastic surgery and oral surgery, but there is limited experience in gynecology and obstetrics (Alcaraz *et al.*, 2015; Borrione *et al.*, 2010; Patel *et al.*, 2016; Yu *et al.*, 2011).

For the first time, Chang reported the efficacy of intrauterine infusion of PRP for endometrial growth in women with thin endometrium. In that trial, PRP was infused in 5 women with inadequate endometrium who had poor response to conventional therapy during the FET cycle. The proper response to treatment was reported in all of them, and normal pregnancy was reported in 4 women (Chang *et al.*, 2015).

Adequate endometrial thickness is a main factor for implantation and pregnancy. Women with persistent thin endometrium often do not undergo embryo transfer. Several methods have been described for endometrial preparation but there is not any definitive method yet. In recent years, intrauterine infusion of G-CSF has been studied but inconsistent results have been reported. Some researchers reported that G-CSF favors endometrial growth and pregnancy. G-CSF is a cytokine that stimulates neutrophilic granulocyte differentiation and proliferation, it may induce endometrium proliferation and growth, thus improve pregnancy outcome. According to this hypothesis, local infusion of PRP that contains several growth factors and cytokines may improve endometrial growth and receptivity. PRP is collected from autologous blood sample, so in comparison to G-CSF, PRP is more accessible and affordable (Gleicher *et al.*, 2011; Lucena & Moreno-Ortiz, 2013).

The results of our pilot study revealed the efficacy of PRP on endometrial growth. Adequate endometrial growth was found in all the participants after two PRP infusions in all patients who had a history of cycle cancellation due to thin endometrium. At the present, there is limited evidence in this regard. Hence, we suggest further clinical trials in this context. PRP is a safe procedure, with minimal risks of transmission of infectious disease and immunological reactions since it is made from autologous blood samples.

## CONCLUSION

According to this study, it seems that PRP was effective for endometrial growth in patients with thin endometrium.

## Abbreviation

ART: Assisted reproductive technology
PRP: Platelet-rich plasma
FET: Frozen-thawed embryo transfer
G-CSF: Granulocyte-colony stimulating factor
ET: embryo transfer
